# Parametric analysis of an efficient boundary condition to control outlet flow rates in large arterial networks

**DOI:** 10.1038/s41598-022-21923-9

**Published:** 2022-11-09

**Authors:** Sharp C. Y. Lo, Jon W. S. McCullough, Peter V. Coveney

**Affiliations:** 1 The Centre for Computational Science, Department of Chemistry, University College London, London, UK; 2 Advanced Research Computing Centre, University College London, London, UK; 3 Informatics Institute, Faculty of Science, University of Amsterdam, Amsterdam, The Netherlands

**Keywords:** Computational science, Biophysics, Biomedical engineering

## Abstract

Substantial effort is being invested in the creation of a virtual human—a model which will improve our understanding of human physiology and diseases and assist clinicians in the design of personalised medical treatments. A central challenge of achieving blood flow simulations at full-human scale is the development of an efficient and accurate approach to imposing boundary conditions on many outlets. A previous study proposed an efficient method for implementing the two-element Windkessel model to control the flow rate ratios at outlets. Here we clarify the general role of the resistance and capacitance in this approach and conduct a parametric sweep to examine how to choose their values for complex geometries. We show that the error of the flow rate ratios decreases exponentially as the resistance increases. The errors fall below 4% in a simple five-outlets model and 7% in a human artery model comprising ten outlets. Moreover, the flow rate ratios converge faster and suffer from weaker fluctuations as the capacitance decreases. Our findings also establish constraints on the parameters controlling the numerical stability of the simulations. The findings from this work are directly applicable to larger and more complex vascular domains encountered at full-human scale.

## Introduction

In recent years, the central goal of computational biomedicine to create a virtual human, also referred to as a human digital twin, has become increasingly tangible^1–4^. A virtual human is a detailed, digital representation of an individual’s biophysical processes^5,6^. Such a model will improve our understanding of human biology and pathology and assist clinicians in the design of personalised medical treatments. The development of a virtual human is an ongoing endeavour which requires many computational and algorithmic developments to accurately and efficiently capture human biology at all scales. This paper aims to bring us closer to achieving high-fidelity simulation of blood flow at full-human scale.

When studying a subset of an arterial network, the vessels beyond the smaller system are often truncated to reduce computational cost while boundary conditions are imposed at the inlets and outlets to close the system. A central challenge is the development of an efficient and accurate approach to impose boundary conditions on many outlets. This problem has received substantial interest^7–13^. The boundary conditions should accurately represent the flow in the truncated vessels as they generally affect the entire simulation domain^14^. In particular, outlet boundary conditions have greater influence on the internal flow than inlet ones^15^.

An accurate type of outlet boundary condition specifies the coupling of the simulation domain with so-called Windkessel models. These models relate the blood pressure (*P*) and the volumetric flow rate (*Q*) at the domain outlets to the interaction between the arterial compliance, peripheral resistance, and flow inertia in the truncated vessels^16–18^. While they have superior accuracy compared with other boundary conditions^19–21^, they suffer from the high cost in calibration required due to the large number of model parameters involved.

Grinberg and Karniadakis^8^ proposed an efficient strategy for the calibration concerning the two-element Windkessel (WK2) model, the simplest one-compartment description composed of a resistor and a capacitor^17,18^. They showed that the *Q* ratios between the outlets are inversely proportional to the resistance (*R*) in the WK2 models under certain conditions, and because of this, the *Q* ratios can be controlled by tuning one single parameter. This explicit and simple relation renders the strategy efficient when applied to large arterial networks and easy to implement. Nevertheless, it is difficult to articulate this approach in the context of irregular domains. In addition, the role of the capacitance (*C*) in the strategy and how to choose its value in practice remain unanswered.

To relax these limitations, here we extend the theoretical framework of the strategy to more complex flows and study the effects of *R* and *C* on the accuracy of the *Q* ratios, the convergence and fluctuation of the flow variables, as well as the stability of the simulations. We conduct this study by a parametric sweep over the values of *R* and *C* in the simulations of fluid flows in two irregular domains solved by the lattice Boltzmann method (LBM)^14^. The additional understanding from our findings assists users of the approach in their selection of the model parameters for flows in complex geometries.

## Methods

In this section, we describe the set-up of the numerical experiments performed in this study. We first introduce the implementation of the two-element Windkessel model. Next, we explain how we determine the model parameters and the desired flow rate ratios. After that, we address some implementation details. Lastly, we describe the two simulation domains used.

### The two-element Windkessel model

The WK2 model is comprised of a resistor and a capacitor connected in parallel^17,18^. The resistor describes the dissipation of small peripheral vessels including arterioles and capillaries, whereas the capacitor describes the storage properties of large arteries. This model has been used to capture some important features of the pulse waves in arterial networks^22–25^.

The strategy proposed by Grinberg and Karniadakis^8^ is based on the use of the WK2 model as the outlet boundary conditions. These boundary conditions are implemented as follows. At each outlet, the flow rate, *Q*, is first calculated by integrating the flow velocity $\mathbf {U}$ obtained during the simulation over the boundary plane:

1 $$Q = \int \mathbf {U} \cdot \hat{\mathbf{n}} \,\, dA,$$

where $\hat{\mathbf{n}}$ and *A* are, respectively, the normal vector and the area of the boundary plane pointing outwards from the domain. The pressure, *P*, is obtained by solving the differential equation

2 $$\begin{aligned} P + RC \frac{dP}{dt} = RQ. \end{aligned}$$

Here *R* and *C* are the resistance and the capacitance of the WK2 model respectively. Lastly, the outlet is imposed by a pressure condition with the calculated value of *P*. To solve Eq. (2) numerically, we discretise it with a semi-implicit scheme^8^. One can alternatively use an explicit scheme^26^. Hence, we obtain

3 $$\begin{aligned} P^{n+1} = \frac{RC}{RC + \Delta t} P^{n} + \frac{R \Delta t}{RC + \Delta t} Q^{n}, \end{aligned}$$

where $\Delta t$ is the time step size and *n* is the index of the time step.

### Choice of resistance

An attractive feature of the WK2 model is that the flow rate of multiple outlets can be set to the desired ratios by tuning one single parameter. Provided that *R* is sufficiently large at all outlets, the ratio of the flow rate in an arbitrary outlet *j* to that in the reference outlet *ref* satisfies the *Q*–*R* relation^8^:

4 $$\begin{aligned} \frac{Q_\text {ref}}{Q_j} \approx \frac{R_j}{R_\text {ref}} \equiv \eta _j, \end{aligned}$$

where $j = 1, \ldots , N$ if there are *N* outlets. The ratios between $R_j$ are determined by the desired *Q* ratios, whereas the magnitudes of $R_j$ are controlled by $R_\text {ref}$, the unique free parameter. It is possible to impose time-dependent *Q* ratios in order to incorporate in vivo or in vitro flow data^8^. In this study, we consider the time-independent case and choose the reference outlet to be the one with the largest *Q* so that $\eta _j \ge 1$.

The key step of this strategy is to find an $R_\text {ref}$ such that $R_j$ is sufficiently large for all *j*. The condition for Poiseuille flow was derived^8^ to be $R_j \gg L_j K_j$ for all *j*. Here *L* is the length of the cylindrical pipe that the WK2 model is coupled with, and *K* is the flow resistance per length given by

5 $$\begin{aligned} K = \frac{8 \mu }{\pi r^{4}}, \end{aligned}$$

where $\mu$ is the dynamic viscosity of the fluid and *r* is the radius of the pipe. However, the conditions for such flow in complex geometries have rarely been studied directly. In broad terms, this problem can be addressed as follows. A vessel of arbitrary shape can be conceived as a cylindrical pipe of length *L* and flow resistance per length *K* such that *LK* is equal to the total flow resistance of the vessel. When the vessel is coupled with the WK2 model, the overall flow resistance is $LK + R$. Hence, a necessary condition for the *Q*–*R* relation to hold must be $R \gg LK$ at all outlets; otherwise, *LK* would appear in Eq. (4).

It is tempting to choose a very large $R_\text {ref}$ to ensure $R \gg LK$ at the outlets, but this is not always feasible as the simulation may not stabilise; we show that in the Results section. Moreover, it is of interest to know the lower limit of $R_\text {ref}$ for which the simulated *Q* ratios admit an acceptable error. To tackle this problem, we obtain a conservative estimate of the largest value of *LK* among the outlets, denoted by $\hat{L}\hat{K}$, and assess different multiples of $\hat{L}\hat{K}$ for $R_\text {ref}$ in the following way.

Let us first describe the estimation of $\hat{L}\hat{K}$. In view of Eq. (5), the product *LK* has a stronger dependency on *r* than *L*. Therefore, when selecting vessels for the estimation, *r* should be prioritised over *L*. To simplify the procedure, we use only the outlet vessel with the smallest *r* for the estimation. Here *r* is the equivalent radius of the circle that has the same area as the boundary plane; this area is obtained during the voxelisation of the geometry^27^. By substituting *r* into Eq. (5), $\hat{K}$ is estimated. The length $\hat{L}$ is approximated by the distance between the bifurcation point and the boundary plane of the vessel. We note that since a circle has the largest area among all the shapes of the same perimeter, using the equivalent radius gives the most conservative estimate and therefore $\hat{K}$ will not be underestimated. For a more accurate estimation of $\hat{K}$, one can apply the formulae for boundary planes of special shapes^28^.

We expect that the simulated *Q* ratios will be accurate only if $R_\text {ref} \ge \hat{L}\hat{K}$. We denote the multiple of the lower limit by $\gamma _R$ so that $R_\text {ref} = \gamma _R \hat{L}\hat{K}$ in a simulation. By performing simulations with different values of $\gamma _R$, we study the effects of *R* on the accuracy of the simulated *Q* ratios.

### Choice of capacitance

Grinberg and Karniadakis^8^ showed that *C* is responsible for damping the high-frequency waves in the simulation. This is an advantage for vascular simulations as high-frequency waves are not significant in blood flow^29^. They reported that choosing *C* such that $RC = \mathcal {O}(1)$ was favourable for their simulations. However, we find that such a choice is not suitable for simulations in general as it leads to instability in our simulations.

Indeed, the transient period at the beginning of the simulation is essential to the stability of the simulation. The transient period can be attributed to the solution of the homogeneous part of Eq. (2), i.e. $P(t) = P(0) \exp (-t / RC)$. This solution has a characteristic decay time of *RC*^8^. Although it will fade out eventually, it can exhibit an immense magnitude at the beginning of the simulation. As a result, certain stability conditions may be violated, causing the simulation to become unstable. Therefore, the choice of *C* should be based on a suitable value of *RC*.

Since *RC* has dimensions of time, a natural choice is the order of the fundamental frequency ($\omega _0$) of the flow being simulated. For example, a heartbeat with a period of *T* = 0.8 s has $\omega _0 = 2 \pi / T$= 7.85 s^-1^. As the transient solution is not the target of the WK2 model, it should fade out in a reasonably short time. Therefore, $RC \gg 1 / \omega _0$ should be avoided. This provides an upper limit to the appropriate range of *RC*. On the other hand, the lower limit can be found from the perspective of the frequency domain. By taking the Fourier transform of Eq. (2), we obtain

6 $$\begin{aligned} \hat{P}(\omega )&= Z(\omega ) \hat{Q}(\omega ), \end{aligned}$$

7 $$\begin{aligned} Z(\omega )&= \frac{R}{1 + i \omega RC} = \frac{R}{\sqrt{1 + (\omega RC)^2}} \exp [i \tan ^{-1}(- \omega RC)], \end{aligned}$$

where $\omega$ is the angular frequency of a Fourier mode, *Z* is the impedance of the circuit analogue^18^ of the WK2 model, and $i = \sqrt{-1}$. According to Eq. (7), the phase difference between $\hat{P}(\omega )$ and $\hat{Q}(\omega )$ is given by $\tan ^{-1} (- \omega RC)$. This implies a time lag of *Q*(*t*) from *P*(*t*) in the time domain which decreases as *RC* approaches 0. Such time lag has been observed in several experiments^8,29,30^. To comply with these observations, $RC \ll 1 / \omega _0$ should be avoided, which in turn gives the lower limit.

Although the expected range of *RC* may prove unfeasible due to practical constraints such as stability, it circumscribes the range within which a suitable value of *C* can be found. Based on that range, we express *C* in an outlet *j* in terms of the parameter $\gamma _{RC}$ such that $C_j = \gamma _{RC} / (\omega _0 R_j)$. We use the same value of $R_j C_j$ for all *j* to make the decay time scale consistent. In other words, $\gamma _{RC}$ governs the value of *C* one should select for a given *R*. By varying the value of $\gamma _{RC}$ used in the simulations, we can locate an optimal value of *C* for a given *R*.

### Desired flow rate ratios

One of the goals of this work is to demonstrate the applicability of our methods to simulations of actual medical importance, so we need *Q* ratios which reflect a physiological flow in the geometry. Based on the principle of minimum work, Murray^31^ showed that *Q* is proportional to $r^3$ in any section of a vessel. Invoking mass conservation, Murray’s law can be written as^21^

8 $$\begin{aligned} Q_j = \frac{r_j^3}{\sum _j r_j^3}, \end{aligned}$$

where *j* is the index of an outlet. It is known that Murray’s law does not hold true in general. For a more accurate description of the relation between *Q* and *r*, other powers of *r* can be used^21,32^.

Here we use the equivalent radius of the outlet vessels (see Choice of Resistance) for $r_j$ to compute $Q_j$. By substituting $Q_j$ into Eq. (4), we obtain the ratios of the resistances, $\eta _j$, or equivalently the inverse of the desired *Q* ratios. This method is useful when data on physiological *Q* ratios is unavailable because only knowledge about the geometry is required. The voxeliser we use^27^ calculates the area of the outlets systematically and hence the equivalent radii of the outlet vessels. This allows us to apply the strategy efficiently when the geometry contains many outlets.

### Model of internal flow

The LBM^14^ is attractive for haemodynamic simulations as it is highly scalable^33,34^ due to data locality and its applicability to complex geometries. We simulate the blood flow using HemeLB^35,36^, which is an open-source fluid flow solver based on the LBM. We use the D3Q19 velocity set and the Bhatnagar-Gross-Krook collision operator as the LBM model for the three-dimensional blood flow^37^. We assume the vessels wall to be rigid with the use of the Bouzidi–Firdaouss–Lallemand wall boundary condition^38^. This assumption can be removed by using the elastic wall boundary condition available in HemeLB^39^. The solver describes the blood as a Newtonian fluid, while other rheology models are also available^40,41^.

In this work, a simulation is considered unstable if any single particle distribution function of the LBM in the domain becomes non-positive and stable otherwise^14^. To ensure the compressibility error is small, the relaxation time of the collision operator is chosen such that the Mach number is maintained below 0.1 throughout the simulations^14^.

### Inlet boundary condition

For the inlet, we impose a velocity boundary condition using Ladd’s method^38,42^. We assume that $\mathbf {U}$ at the inlet is normal to the boundary plane. Hence, $\mathbf {U}$ can be separated into spatial and temporal parts as $\mathbf {U}(\rho ,t) = f(\rho ) \ \mathbf {U}_{\max }(t)$, where *f* is a function of the radial distance $\rho$ from the geometric centre $\rho _{\max }$ and $\mathbf {U}_{\max }$ is the velocity at the centre. The separation has the advantage that $\mathbf {U}$ can be assigned before the simulation begins as $f(\rho )$ depends solely on the geometry of the boundary plane. Regarding the spatial profile, we use the quadratic form pertaining to Poiseuille flow:

9 $$\begin{aligned} f(\rho ) = 1 - \frac{\rho ^2}{\rho ^2_{\max }}, \end{aligned}$$

which is valid if the flow is quasi-static and laminar. It can be applied to boundary planes which are circular, where $f(\rho )$ becomes a parabola, as well as those of irregular shapes^43^. Regarding the temporal profile, we impose a sinusoidal wave of frequency $\omega$ on top of a mean flow $\mathbf {U}_{\text {mean}}$ such that $\mathbf {U}_{\max }(t) = \mathbf {U}_\text {mean} [1 + 0.1 \cos (\omega t)]$. The values of $\mathbf {U}_\text {mean}$ and $\omega$ are calculated from the Womersley number, given by $r\sqrt{\omega /\nu }$, and the maximum Reynolds number, given by $2 \big\Vert \mathbf {U}_{\max }(0) \big\Vert r/\nu$ at the inlet, where $\nu$ is the kinematic viscosity of the fluid. In order that the spatial profile is valid in our simulations, we choose 2 and 10 for these numbers respectively.

### Techniques to exploit data locality and adapt for complex geometries

The computation of *Q* at the outlets in Eq. (1) requires data from all the boundary lattice sites which hinders the data locality feature of the LBM, and to overcome this we use the following method. We assume that $\mathbf {U}$ is normal to the boundary plane, i.e. $\mathbf {U} \cdot \hat{\mathbf{n}} = \big\Vert \mathbf {U} \big\Vert$, which is valid since the Reynolds number is small in our simulations. We further assume that $\mathbf {U}$ exhibits the profile $f(\rho )$ in Eq. (9) as for the inlet, which is valid since the Womersley number is even smaller in the outlets than the inlet. Hence, Eq. (1) can be written as

10 $$\begin{aligned} Q(t) = \big\Vert \mathbf {U}(\rho ,t) \big\Vert \ \frac{\int f(\rho ) \ dA}{f(\rho )}. \end{aligned}$$

In this equation, the fraction can be computed and stored locally before the simulation begins as it depends solely on the outlet geometry. Besides, $\mathbf {U}(\rho ,t)$ can be obtained locally in the context of LBM. As a result, the data locality feature of the LBM can be exploited.

To implement the pressure condition in the WK2 model, we adopt the method of Nash et al.^38^ which is suitable for complex geometries. This method assumes that *P* is uniform over an outlet plane. A boundary lattice site close to the centre of the outlet plane is used for updating *P* during the simulation.

### Simulation domains

Two geometries are used as the simulation domain in our experiments. The first geometry is a five-outlets model which has a more regular shape. The other is a human artery model of a more complex shape. These models are publicly available at 10.5522/04/c.6223232.v1.

#### Five-outlets model

**Figure 1:**
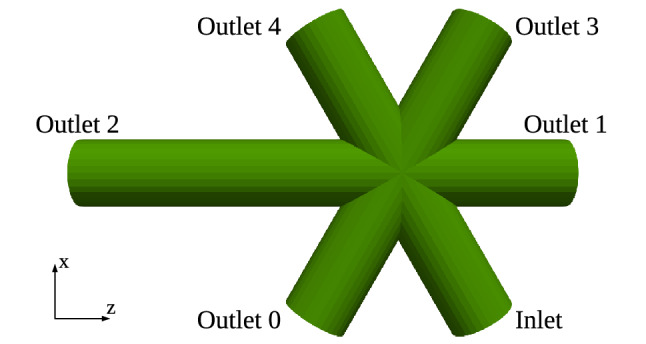
The five-outlets model in the lateral view. It is composed by joining five identical short pipes and a longer pipe all converging on a single point. The longer pipe is twice the length of a short pipe.

The five-outlets model, as shown in Fig. 1, is composed by joining five identical short pipes and a longer pipe all converging on a single point. It is a simplified model of the aortic arch with five branches reported by Ma et al.^44^. The short pipes have a length of 4.83 mm, and the longer pipe is twice the length of a short pipe. All of them are circular with a uniform radius of 1 mm and have an open boundary at their ends. One of the short pipes is a fluid inlet, whereas the others are fluid outlets. The geometry is voxelised^27^ to the three resolutions 80 µm, 40 µm, and 20 µm such that there are about 13, 25, and 50 lattice sites along any pipe radii respectively.

This geometry is used to confirm the role of the various parameters described above with a focus on numerical aspects. Indeed, the desired *Q* ratios of the outlets can be chosen regardless of physiology. The ratios for outlets 0 to 4 are set accordingly to be 3 : 4 : 5 : 6 : 7, translating into $\eta _j = \frac{7}{3}, \frac{7}{4}, \frac{7}{5}, \frac{7}{6}, 1$ for $j = 0,1,2,3,4$ respectively. The relaxation time of the LBM model is set to be 0.8 for the coarse grid and 0.9082 for the other two grids, both in lattice units.

#### Profunda femoris model

**Figure 2:**
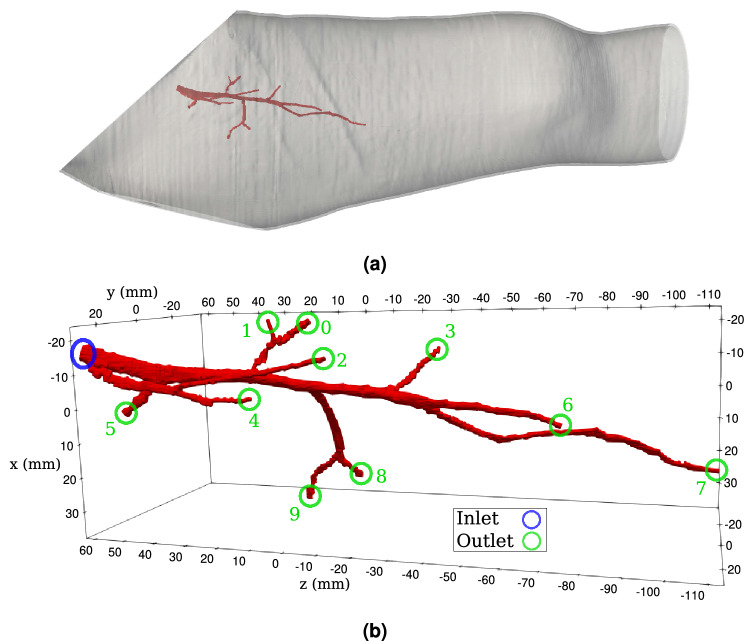
The *profunda femoris* model used in this study is constructed from the *profunda femoris* artery on the right thigh of a human subject, as shown in **(a)**. The location of the inlet and the outlets are indicated in **(b)** with the index of the outlets labelled.

Another geometry is a model of an artery in the right thigh of humans, called the *profunda femoris* model (see Fig. 2). It is extracted from the computational anatomical model used in our previous work^1,45^. To meet the assumptions of the pressure BC used in our simulations, we modify some of the outlets such that the normal of the outlet planes is closer to the flow direction. This geometry is chosen for the following reasons: (i) it is a complex domain in which the vessels have arbitrary shapes; (ii) second, Murray’s law should be valid since the geometry is constructed from a human model; (iii) third, the number of outlets, being 10, is already substantial; (iv) fourth, complications arising from imposing multiple inlet boundary conditions are avoided given that our focus is on the outlets.

The equivalent radius, *r*, of the inlet plane is 1.67 mm, whereas that of the outlet planes range from 0.50 mm at outlet 8 to 1.12 mm at outlet 5. The outlet vessel with the smallest *r* has a length of 8.74 mm. The geometry is voxelised^27^ to the three different resolutions 49 µm, 35 µm, and 25 µm such that there are about 10, 14, and 20 lattice sites along the equivalent radius of the narrowest vessel respectively. This results in around 16.3 M, 44.6 M, and 122 M lattice sites in the entire domains respectively. The desired *Q* ratios are obtained by applying Murray’s law in Eq. (8). The resulting ratios of the resistances are $\eta _j = 3.17:8.71:6.25:1.65:10.7:1:3.15:4.80:13.0:5.81$. The relaxation time of the LBM model is set to be 0.9082 in lattice units for all the grids.

## Results

In this section, we first present the results from the simulations on the five-outlets domain and later those on the *profunda femoris* domain. The simulations were performed using 48 to 4608 Intel Xeon Platinum 8174 Processors on SuperMUC-NG at the Leibniz Supercomputing Centre. The analysis tools and parameters used to obtain the results are publicly available at 10.5522/04/c.6223232.v1.

### Simulations on the five-outlets domain

We will study different aspects of the simulation results: stability of the simulations, accuracy of the *Q* ratios, convergence and fluctuation of the flow variables.

#### Stability maps

**Figure 3:**
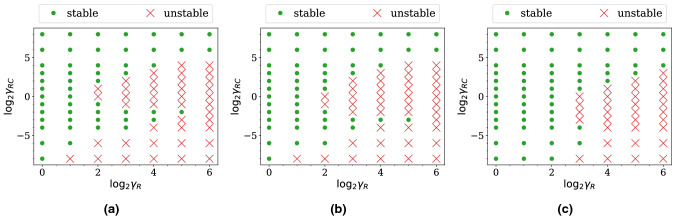
Stability map of the simulations on **(a)** the coarse grid, **(b)** the medium grid, and **(c)** the fine grid of the five-outlet domain. The three maps reveal the same pattern: for $\log _2 \gamma _{RC} \ge 0$, a larger $\gamma _{RC}$ allows a larger $\gamma _R$; for $\log _2 \gamma _{RC} \le -3$, a smaller $\gamma _{RC}$ requires a smaller $\gamma _R$.

As mentioned, the simulations we perform do not admit an arbitrary choice of *R* and *C* due to numerical instabilities in the flow solver. To investigate what choices lead to stable simulations, we run simulations on the five-outlets domain with different combinations of $\gamma _R$ and $\gamma _{RC}$ for one period of the inlet flow. The duration of one period is sufficient since instability always occurs within this period in these simulations. We display a stability map for each resolution (see Fig. 3) showing what combinations lead to a stable or an unstable simulation. Comparing between the three maps, we find that a finer grid can admit a larger collection of the parameters than a coarser grid. Nonetheless, all of them reveal the same pattern: for $\log _2 \gamma _{RC} \ge 0$, a larger $\gamma _{RC}$ allows a larger $\gamma _R$; for $\log _2 \gamma _{RC} \le -3$, a smaller $\gamma _{RC}$ requires a smaller $\gamma _R$.

#### Error of flow rate ratios

Since it is not always possible to use an arbitrarily large *R* to achieve the desired *Q* ratios, we need to understand how the error of the simulated *Q* ratios changes with *R* and also *C*. We select a subset of cases from the stability maps and perform simulations for 10 periods of the inlet flow. The accuracy of the simulated *Q* ratios is evaluated by comparing their temporal average in the stationary state with the desired *Q* ratios; the average covers the fourth to the tenth period.

**Figure 4:**
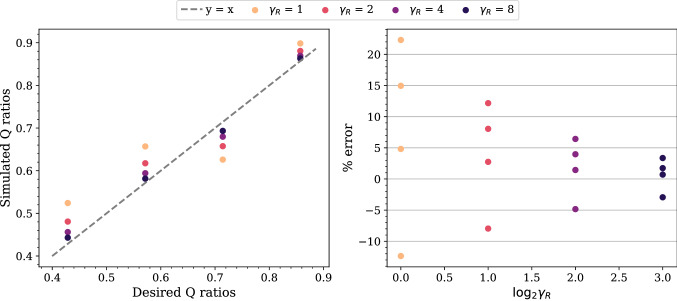
Comparison between the simulated and the desired flow rate (*Q*) ratios as $\gamma _R$ varies for a fixed $\gamma _{RC}/\gamma _R$. The results are obtained from the simulations on the fine grid of the five-outlets domain using $\gamma _{RC}/\gamma _R = 1$. With the outlet 4 arbitrarily chosen as a reference, the *Q* ratios of the other four outlets are obtained for each $\gamma _R$. We observe that the percentage error of the *Q* ratios decreases exponentially as $\gamma _R$ increases.

In all the selected cases, the error of the *Q* ratios at all the outlets decreases exponentially as $\gamma _R$ increases with constant $\gamma _{RC}/\gamma _R$. A comparison between the simulated and the desired values on the fine grid is given in Fig. 4. In this case, the errors are within 4% when $\log _2 \gamma _R = 5$. In addition, we observe that the ratios have no negligible difference when $\gamma _{RC}$ changes with $\gamma _R$ fixed (see Supplementary Fig. S1). The grid resolution has minor impact on the percentage errors (see Supplementary Fig. S3).

#### Convergence rate of flow variables

**Figure 5:**
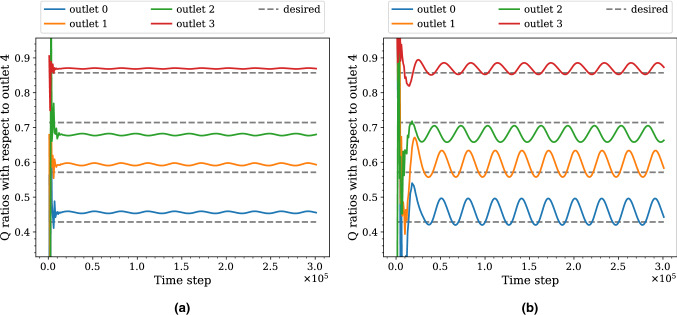
Time series of the flow rate (*Q*) ratios for the outlets in simulations with different $\gamma _{RC}$ but the same $\gamma _R$. The results are obtained from the simulations on the fine grid of the five-outlets domain using **(a)** $\gamma _{RC} = 0.125$ and **(b)** $\gamma _{RC} = 8$ with the same $\gamma _R = 4$. The outlet 4 is used as the reference when computing the ratios. We observe a longer transient period and stronger fluctuations in **(b)** than **(a)**.

**Figure 6:**
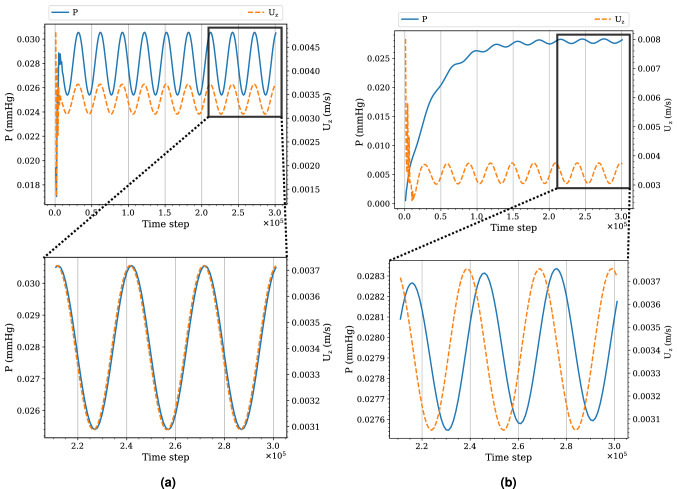
Time series of the pressure (*P*) and the *z*-component of the flow velocity ($U_z$) at the centre of outlet 1 in simulations with different $\gamma _{RC}$ but the same $\gamma _R$. The results are obtained from the simulations on the fine grid of the five-outlets domain using **(a)** $\gamma _{RC} = 0.125$ and **(b)** $\gamma _{RC} = 8$ with the same $\gamma _R = 4$. We observe that both *P* and $U_z$ converge faster in **(b)** than **(a)**; the phase lag of $U_z$ from *P* is also larger in **(b)** than **(a)**.

Since $\gamma _{RC}$ is proportional to the time scale *RC*, we expect it to affect the convergence of the flow variables. Indeed, the *Q* ratios take a longer time to converge to the stationary state with a larger $\gamma _{RC}$ for a given $\gamma _R$, as demonstrated in Fig. 5. In the figure, the *Q* ratios change irregularly at the beginning of both simulations which is the transient period mentioned above. The effect on the convergence rate also applies to *P* and $\mathbf{U}$ on the boundary planes, as can be observed in the upper panel of Fig. 6.

The convergence rate is found to be highest for the *Q* ratios, intermediate for $\mathbf{U}$, and lowest for *P* for a given $\gamma _R$ and $\gamma _{RC}$. Using $\gamma _R = 4, \gamma _{RC} = 8$ on the fine grid as an example, while the *Q* ratios are nearly stationary after 30,000 time steps (see Fig. 5b), $\mathbf{U}$ and *P* still exhibit observable change after 60,000 and 120,000 time steps respectively (see Fig. 6b). Here 30,000 time steps correspond to about one period of the inlet flow.

#### Fluctuation of flow rate ratios

When comparing between the two cases in Fig. 5, we also observe differences in the amplitude of the fluctuations. We observe stronger fluctuation in the *Q* ratios as $\gamma _{RC}$ increases with $\gamma _R$ fixed. Since *Q* is the integration of $\mathbf{U}$ and the ratios between outlets reflect the extent of synchronisation, this phenomenon implies that $\mathbf{U}$ at different outlets is more asynchronous as $\gamma _{RC}$ increases. This in turn suggests that we look at the phase difference between *P* and $\mathbf{U}$. Indeed, as shown in the lower panel of Fig. 6, the phase difference increases with $\gamma _{RC}$.

### Simulations on the profunda femoris domain

This subsection presents the results obtained from the simulations on the *profunda femoris* domain. Here we address the stability of the simulations, the convergence rate and the error of the *Q* ratios.

#### Stability maps

**Figure 7:**
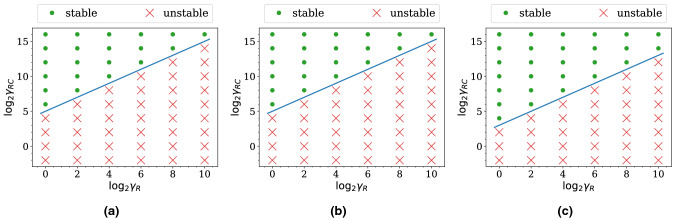
Stability map of the simulations on **(a)** the coarse grid, **(b)** the medium grid, and **(c)** the fine grid of the *profunda femoris* domain. Notably, the stability and the instability region are separated by a straight line of slope 1 in the maps. The *y*-intercept of the line is the smallest for the fine grid.

We first run simulations with different combinations of $\gamma _R$ and $\gamma _{RC}$ for one period of the inlet flow to obtain the stability maps. The maps for the three resolutions are plotted in Fig. 7. They show that a larger $\gamma _R$ requires a larger $\gamma _{RC}$ for the simulation to be stable. Furthermore, the stable and the unstable regions are separated by a straight line of slope 1. By using the definition of $\gamma _{RC}$ and $\gamma _R$ we find that the line corresponds to a constant *C*. The constant is smaller if the *y*-intercept is smaller. Thus, the constant is the smallest in the fine grid case.

#### Convergence rate of flow rate ratios

Next, we select a subset of the stable cases and perform simulations for longer periods of the inlet flow (50 periods for the coarse grid cases and 20 periods for the medium grid cases). In many cases, the *Q* ratios have not reached the stationary state at the end of the simulations. Nevertheless, a smaller $\gamma _{RC}$ for a given $\gamma _R$ leads to faster convergence (see Supplementary Fig. S4). The convergence also increases when $\gamma _R$ gets larger for a given $\gamma _{RC}$ (see Supplementary Fig. S4). The combined effect is that the convergence is faster if the pair of $\gamma _R$ and $\gamma _{RC}$ is closer to the boundary line of the stable region, or equivalently if *C* is smaller. For $\gamma _R = 1024$ and $\gamma _{RC} = 65,536$, the *Q* ratios are nearly stationary after six periods of the inlet flow. We also observe a lower convergence rate in the amplitudes than the means of the *Q* ratios (see Supplementary Fig. S4).

**Figure 8:**
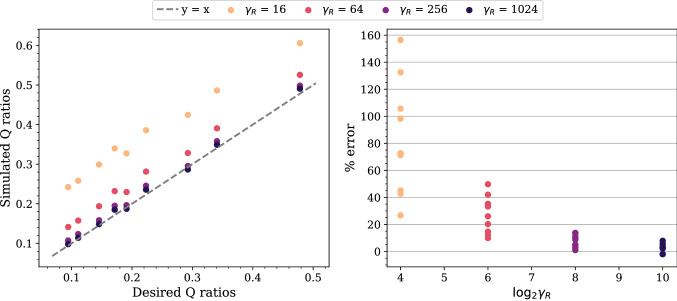
Comparison between the simulated and the desired flow rate (*Q*) ratios as $\gamma _R$ varies for a fixed $\gamma _{RC}/\gamma _R$. The results are obtained from the simulations on the coarse grid of the *profunda femoris* domain using $\gamma _{RC}/\gamma _R = 64$. The outlet 5 was arbitrarily selected as the reference when computing these ratios. We observe that the percentage error of the *Q* ratios reduces exponentially as $\gamma _R$ increases; when $\log _2 \gamma _R = 10$, the errors in all the outlets are within 7%.

#### Error of flow rate ratios

We evaluate the accuracy of the simulated *Q* ratios by using the temporal average of these ratios over the last five periods of the inlet flow. Figure 8 shows the comparison between the simulated and the desired *Q* ratios on the coarse grid. They show that the errors reduce exponentially as $\gamma _R$ increases with a fixed $\gamma _{RC}/\gamma _R$; when $\log _2 \gamma _R = 10$, the errors in all the outlets are within 7%. In addition, we observe that these errors depend slightly on $\gamma _{RC}$ for a given $\gamma _R$ (see Supplementary Fig. S2).

## Discussion

By inspecting the stability map of the simulations, we have found that the higher the grid resolution, the larger the stable region. Moreover, the stable and unstable regions in $\gamma _{RC} \ge 1$ are separated by a line of constant capacitance, *C*, with the stable region being above the line. Since the constant is found to be smaller when a finer grid is used, we can conclude that a higher grid resolution requires a smaller *C* to be stable when $\gamma _{RC}$ is larger than a threshold value.

Our results demonstrate that the flow rate (*Q*) ratios in the simulations approach the desired values as $\gamma _R$ increases. This agrees with the proposed necessary condition for the resistances, *R* (see Choice of Resistance). A further novel finding is that *C* has a little impact on the *Q* ratios, although *C* does not appear in the *Q*–*R* relation in Eq. (4). However, the observed impact may be due to the fact that the *Q* ratios are not completely stationary. Moreover, we have observed that the percentage error of the ratios reduces as the grid resolution increases and that the reduction is more significant in a more complex domain. At the largest $\gamma _R$ and the highest resolution tested, the percentage error of the ratios is within 4% for the five-outlets domain and 7% for the *profunda femoris* domain. We therefore conclude that the *Q*–*R* relation also holds in complex geometries. While testing the *profunda femoris* domain, we observed that the error was highly sensitive to the construction of the outlet shapes. Slight geometric deviations that violated the boundary condition assumptions were observed to generate errors of over 50%.

We have studied the time series of the flow variables in the simulations. The flow variables generally converge more slowly when $\gamma _{RC}$ is larger. This supports the notion that *RC* is the characteristic time scale of the transient period (see “Choice of capacitance”). Another promising finding is that the flow variables converge faster with $\gamma _R$ when $\gamma _{RC} \ge 1$. These observations suggest that a smaller *C* leads to faster convergence of flow variables when $\gamma _{RC}$ is larger than a certain value.

The present findings demonstrate that the *Q* ratios exhibit stronger fluctuations as $\gamma _{RC}$ increases. This can be explained as follows. Suppose there are phase differences in the pressure, *P*, between the outlets. Then a similar pattern can be revealed in *Q* according to Eq. (7). While the phase difference between *Q* and *P* is the same at each outlet because *RC* is constant, it is magnified in a non-linear manner (due to $\tan ^{-1}$ in the equation) as *RC* increases. As a result, the phase differences in *Q* between the outlets will increase with *RC*. These differences manifest as fluctuations in the *Q* ratios.

The above insight enables us to find suitable values of *R* and *C* such that the simulation is stable and the desired *Q*–*R* relation is achieved. To rapidly ascertain if a pair of $\gamma _R$ and $\gamma _{RC}$ can lead to a stable simulation, we can perform simulations on a coarser grid to reduce the computational cost since the stable region for a finer grid is generally larger. If the simulation can stabilise for two different values of $\gamma _R$ but the same value of $\gamma _{RC}$, the larger $\gamma _R$ is preferred since the *Q* ratios will converge faster and be closer to the desired values. If the simulation stabilises for two different values of $\gamma _{RC}$ but the same value of $\gamma _R$, the smaller $\gamma _{RC}$ should be chosen to reduce both the convergence time and the fluctuation of the *Q* ratios.

A limitation of our approach is the long simulation time required. We have shown that the flow variables need a significant time to reach the stationary state. It will be even longer if the desired *Q* ratios are widely spread: the condition $R \gg LK$ at all outlets may make *R* substantial at the outlet with the largest *Q*. Consequently, we will need a large $\gamma _{RC}$ to yield a stable simulation. Then, the flow variables may take a long time to become stationary. According to Murray’s law in Eq. (8), the *Q* ratios have a smaller range if the radii of the outlet vessels have a smaller range. This implies that our method favours vascular models for which the outlet vessels have similar sizes.

The current strategy has limitations in capturing some important flow features due to the use of the WK2 model. For example, the WK2 model may lead to non-physiological reflections of pulse waves because it lacks a resistance connected to the outlet vessel in series^46^. The addition of such a resistance gives rise to the popular three-element Windkessel model^10,15,19,47–50^. We note that such reflections occur in incompressible flows only if the wall motions are considered, but this study does not consider such motions.

While we tested our methods with an inlet flow of single frequency and time-independent *Q* ratios, the methods are designed for more general applications. When the waveform is composed of multiple frequencies, we can choose *C* based on the fundamental frequency of the waveform (see “Choice of capacitance”). When the desired *Q* ratios vary with time, we can use time-varying resistances^8^. The methodology we have described is directly applicable to these situations.

## Conclusions

The strategy proposed by Grinberg and Karniadakis^8^ is attractive for controlling the flow rate (*Q*) ratios of many outlets. Here we study the conditions for this strategy to hold in complex geometries. Based on previous work, we argue that in general the resistance, *R*, in all the outlets should be larger than a threshold value. We propose a method to estimate the threshold and demonstrate it using a simple geometry with five-outlets and a human artery model with ten outlets. We show that the differences between the simulated and the desired *Q* ratios reduce exponentially as *R* increases above the threshold; the differences also reduce mildly as the grid resolution increases. At the largest *R* and the highest grid resolution tested, the differences are within 4% in the five-outlets model and 7% in the human artery model.

Hitherto, there has been limited understanding about the role of the capacitance, *C*, in the strategy as well as how to choose its value in practice. Based on some practical requirements, we derive a suitable order of magnitude for *RC* and parametrise it with $\gamma _{RC}$. By performing simulations with different values of $\gamma _{RC}$, we study the effects of *C* on the flow variables and the stability of the simulations. Our findings show that *C* has some impact on the *Q* ratios, even though the approach is formulated without *C*. Our results reveal that the smaller the value of $\gamma _{RC}$, the faster the convergence rate and the weaker the fluctuation of the flow variables. A further novel finding is that when $\gamma _{RC} \ge 1$ the simulation is stable if *C* is larger than a threshold value.

In conclusion, we have studied the effects of *R* and *C* on the accuracy of the *Q* ratios, the convergence and fluctuation of the flow variables, as well as the stability of the simulations. This additional understanding provides a basis for the calibration of the Windkessel models to achieve a stable simulation and the desired *Q* ratios in the outlets. The methods used in this work are designed for applications where there are many outlets and the inlet profile comprises multiple frequencies. These methods are directly applicable to larger and more complex vascular domains encountered at full-human scale. Future work will investigate the validity of such applications.

## Supplementary Information

Supplementary Figures.

## Data Availability

All data generated or analysed during this study are included in this published article and its supplementary information available at 10.5522/04/c.6223232.v1. The code used for the simulations is publicly available at https://github.com/hemelb-codes/HemePure. The code used for the voxelisation of the geometries is publicly available at https://github.com/UCL-CCS/HemePure_tools.
