# Supplementary material for: Parametric analysis of an efficient boundary condition to control outlet flow rates in large arterial networks
Source: Sci Rep. 2022 Nov 9;12:19092. doi: 10.1038/s41598-022-21923-9 (PMC9646762; doi:10.1038/s41598-022-21923-9)
Supplement: Supplementary file 1 — Supplementary Figures. [file 41598_2022_21923_MOESM1_ESM.pdf]

Supplementary Figures for

Parametric Analysis of an Efficient Boundary Condition to Control Outlet Flow Rates in Large Arterial Networks

Sharp C. Y. Lo[1], Jon W. S. McCullough[1], and Peter V. Coveney[1,2,3,*]

[1]The Centre for Computational Science, Department of Chemistry, University College London, London, United Kingdom
[2]Advanced Research Computing Centre, University College London, London, United Kingdom
[3]Informatics Institute, Faculty of Science, University of Amsterdam, Amsterdam, Netherlands
[*]p.v.coveney@ucl.ac.uk

This document presents additional figures supporting the analysis given in the main text. Here we show the effects of the capacitance (C) and grid resolution on the temporal average of the flow rate (Q) ratios, and the effects of C on the convergence rates of the Q ratios.

Contents

S1 Effects of C on Averaged Q Ratios **2**

S2 Effects of Grid Resolution on Averaged Q Ratios **4**

S3 Effects of C on Convergence Rates of Q Ratios **5**

S1 Effects of C on Averaged Q Ratios

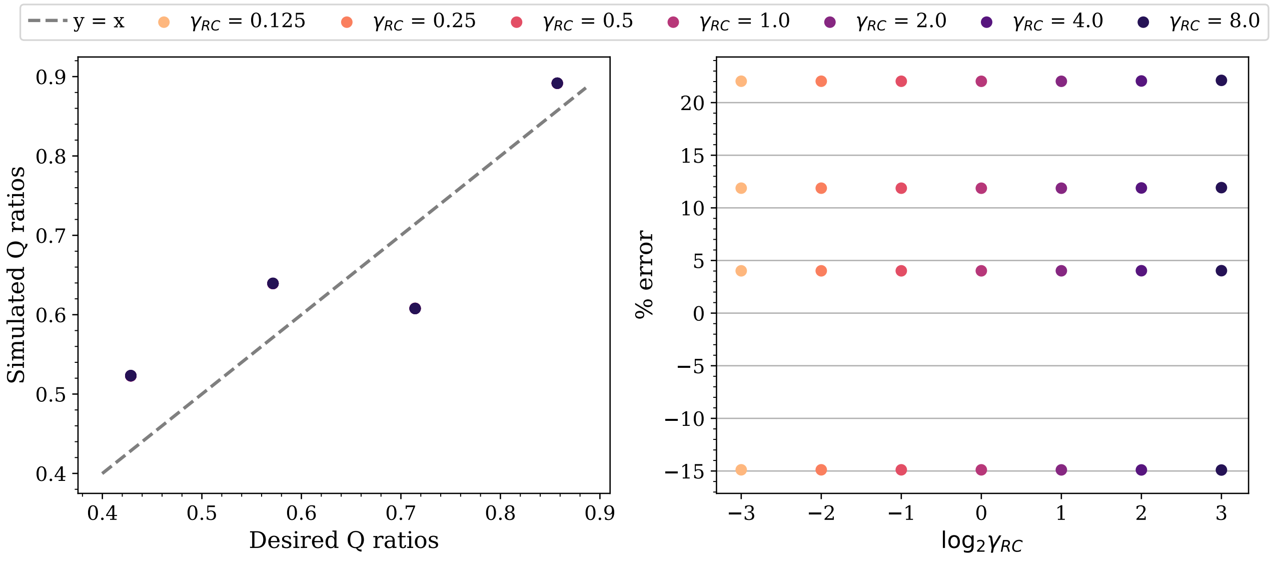

(a)

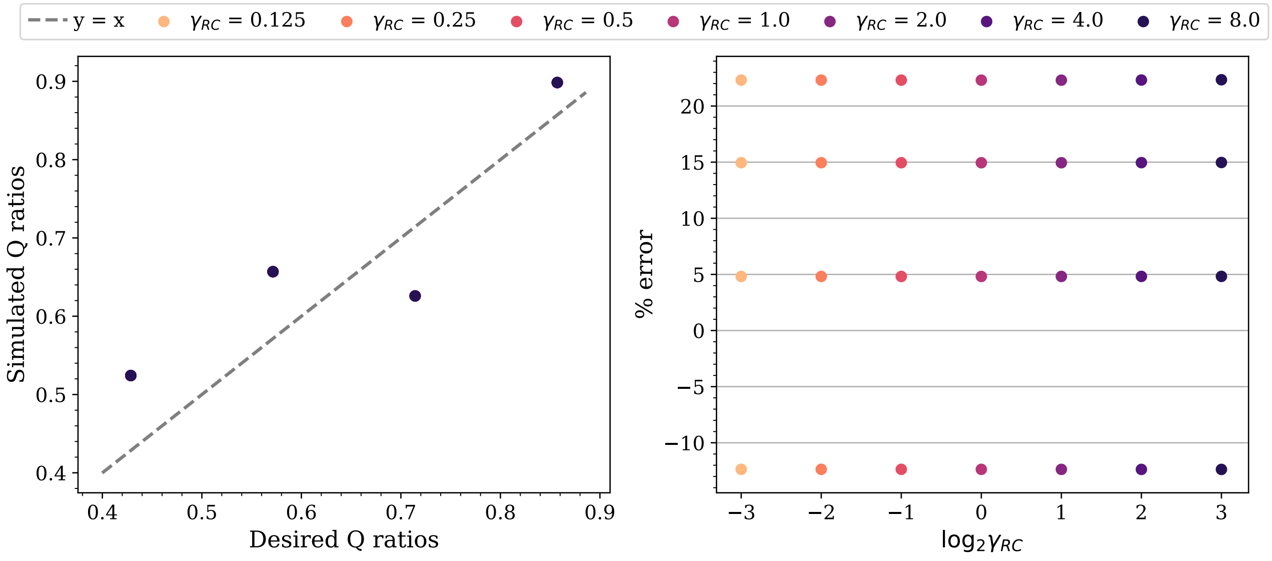

(b)

Figure S1: Comparison between the simulated and the desired flow rate (Q) ratios as γ_{RC} varies for a fixed γ_R. The results are obtained from the simulations on **(a)** the coarse grid and **(b)** the fine grid of the five-outlets domain using $\gamma_R = 1$. The outlet 4 is used as the reference when computing the ratios. We observe that the Q ratios have no significant change with γ_{RC} in both cases.

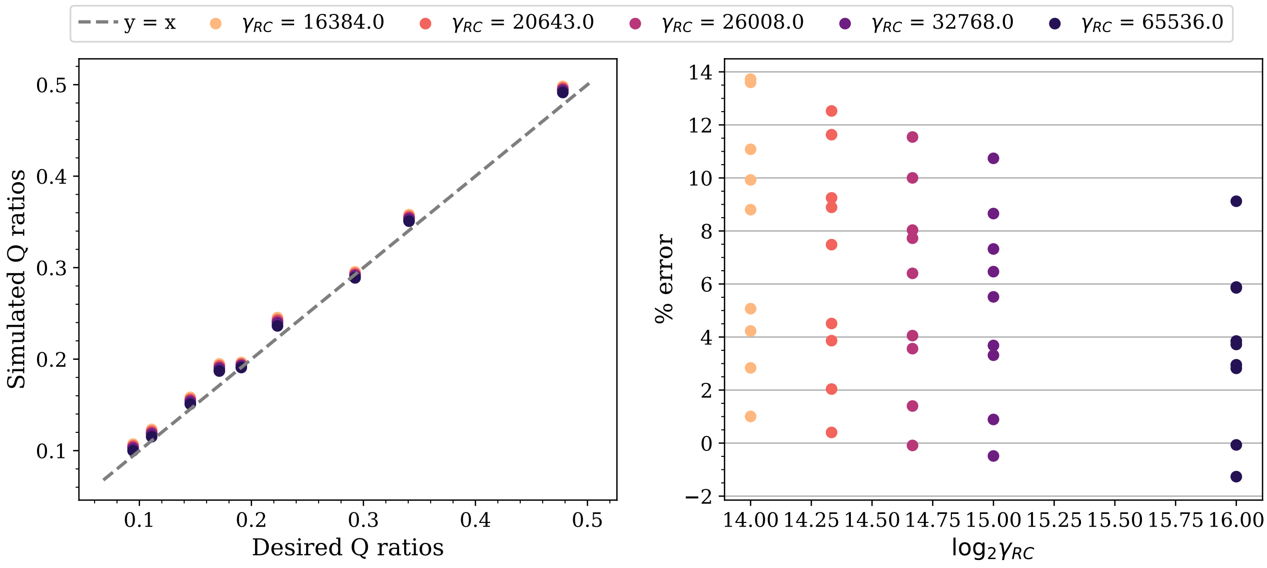

(a)

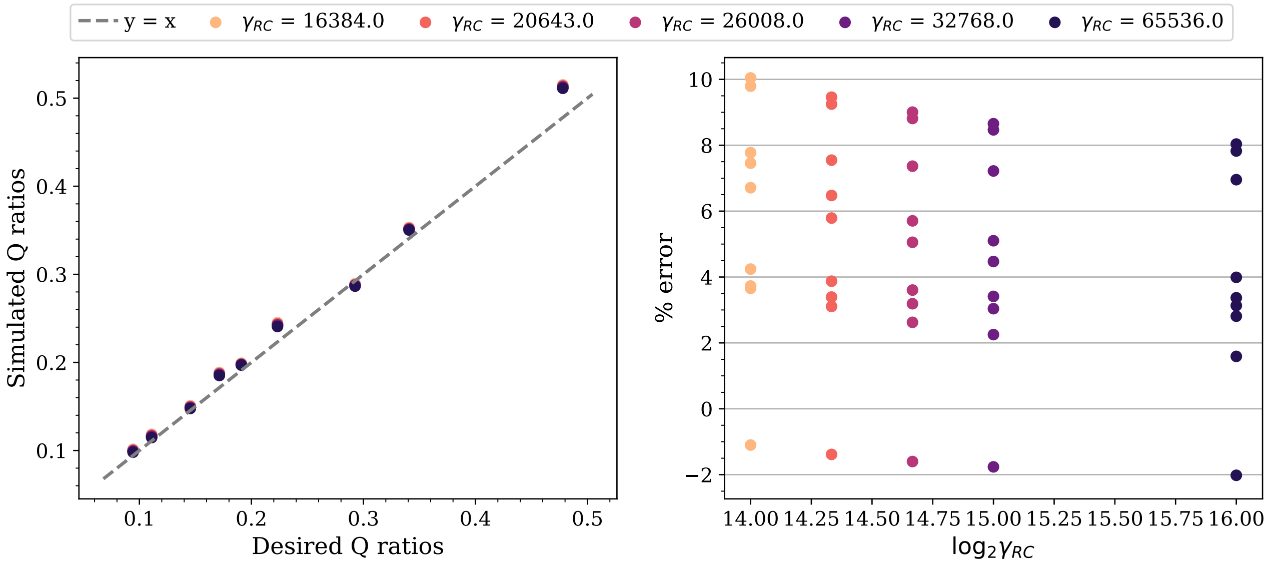

(b)

Figure S2: Comparison between the simulated and the desired flow rate (Q) ratios as γ_{RC} varies for a fixed γ_R. The results are obtained from the simulations on **(a)** the coarse grid and **(b)** the medium grid of the *profunda femoris* domain using $\gamma_R = 256$. The outlet 5 is used as the reference when computing the ratios. We observe that the Q ratios change slightly with γ_{RC}. We attribute this change to the fact that the Q ratios are not completely stationary.

S2 Effects of Grid Resolution on Averaged Q Ratios

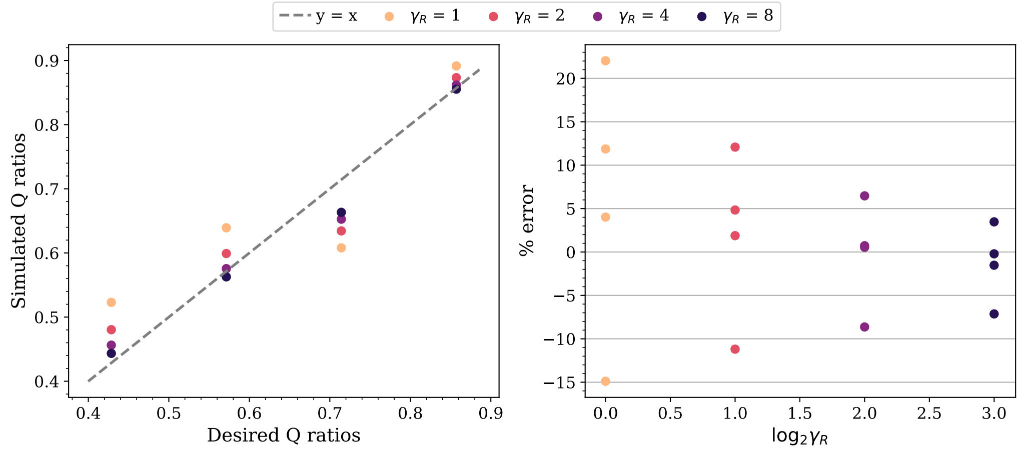

(a)

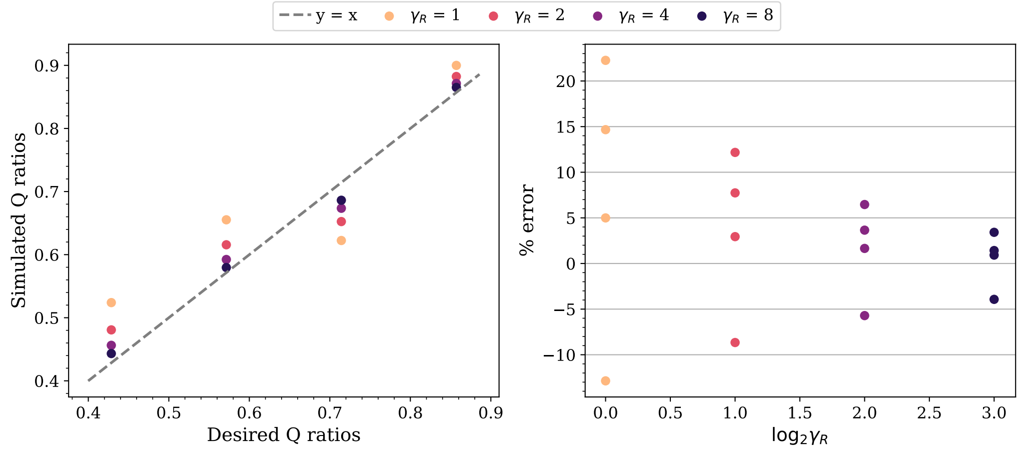

(b)

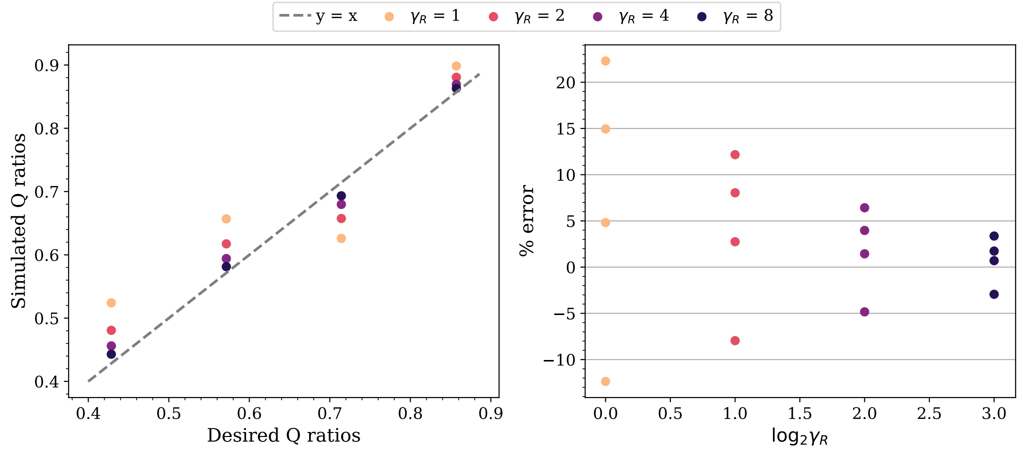

(c)

Figure S3: Comparison between the simulated and the desired flow rate (Q) ratios as γ_R varies for a fixed γ_{RC}/γ_R. The results are obtained from the simulations on **(a)** the coarse grid, **(b)** the medium grid, and **(c)** the fine grid of the five-outlets domain using $\gamma_{RC}/\gamma_R = 1$. The outlet 4 is used as the reference when computing the ratios. The differences in the percentage errors are smaller than 5% between **(a)** and **(b)**, and less than 2% between **(b)** and **(c)**.

S3 Effects of C on Convergence Rates of Q Ratios

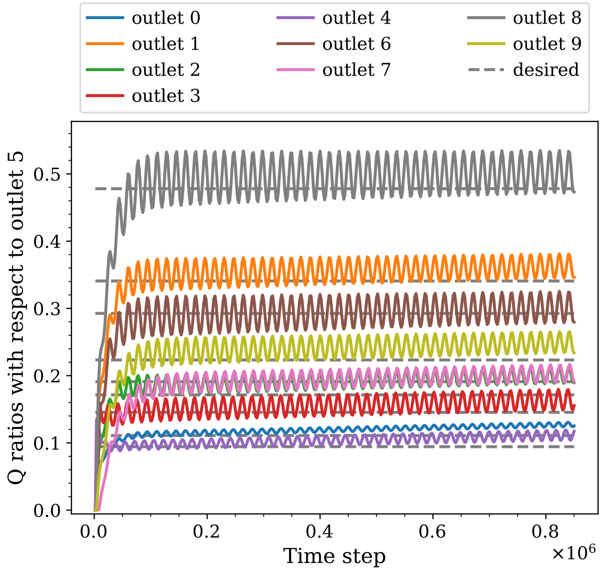

(a) $(\gamma_R, \gamma_{RC}) = (64, 16384)$

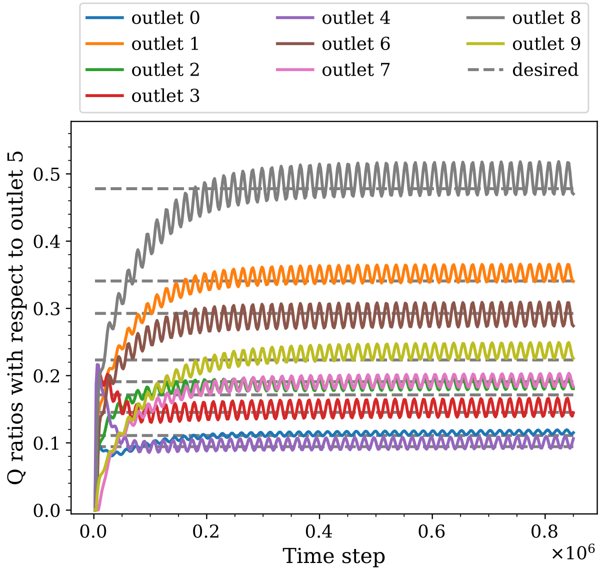

(b) $(\gamma_R, \gamma_{RC}) = (64, 65536)$

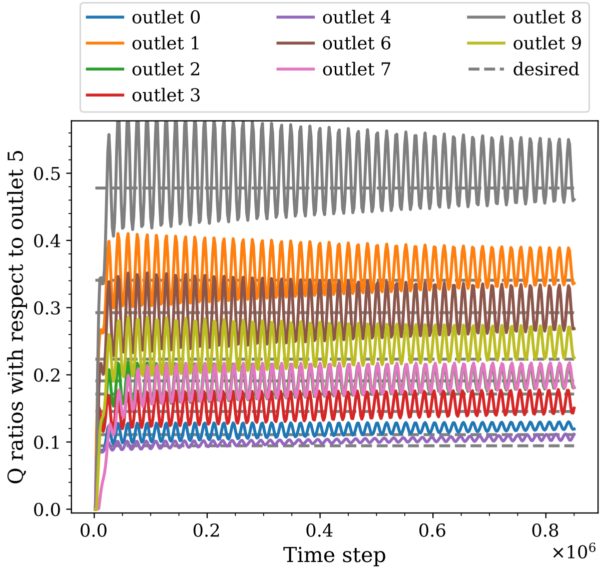

(c) $(\gamma_R, \gamma_{RC}) = (256, 16384)$

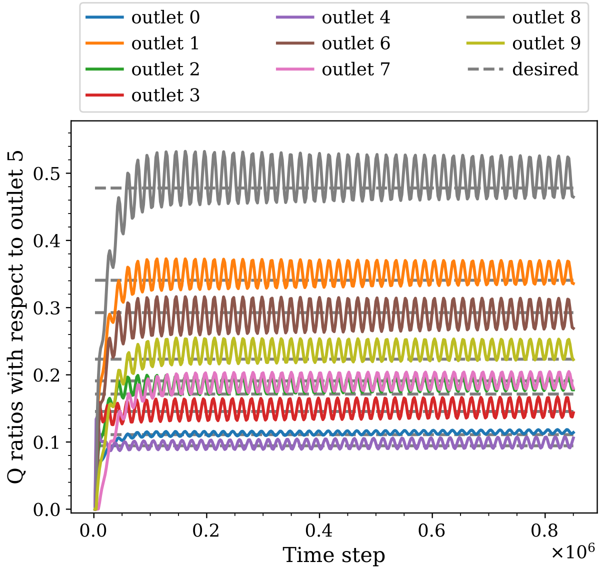

(d) $(\gamma_R, \gamma_{RC}) = (256, 65536)$

Figure S4: Time series of the flow rate (Q) ratios for the outlets in simulations with different sets of (γ_R, γ_{RC}). The results are obtained from the simulations on the coarse grid of the *profunda femoris* domain. The amplitudes of the ratios have not reached the stationary state, whereas the mean of the ratios are nearly steady. The transient period at the beginning of the simulations is shorter for a smaller γ_{RC} with constant γ_R or for a larger γ_R with constant γ_{RC}.
